# Editorial: Childhood leukemias in Latin America: epidemiology, causality, novel predictive profiles and therapeutic strategies

**DOI:** 10.3389/fonc.2024.1509943

**Published:** 2024-11-18

**Authors:** Juan Carlos Núñez-Enríquez, Juan Manuel Mejía-Aranguré, Mario Ernesto Cruz-Muñoz, Rosana Pelayo

**Affiliations:** ^1^ División de Investigación en Salud, Unidad Médica de Alta Especialidad (UMAE) Hospital de Pediatría “Dr. Silvestre Frenk Freund”, Centro Médico Nacional Siglo XXI, Instituto Mexicano del Seguro Social, Mexico City, Mexico; ^2^ Facultad de Medicina, Universidad Nacional Autónoma de México, Universidad Nacional Autónoma de México (UNAM), Mexico City, Mexico; ^3^ Laboratorio de Genómica Funcional del Cáncer, Instituto Nacional de Medicina Genómica, Instituto Nacional de Medicina Genómica (INMEGEN), Mexico City, Mexico; ^4^ Laboratorio de Inmunología Molecular, Facultad de Medicina, Universidad Autónoma del Estado de Morelos, Cuernavaca, Morelos, Mexico; ^5^ Unidad de Educación e Investigación, Instituto Mexicano del Seguro Social, Mexico City, Mexico

**Keywords:** childhood leukemia, Latin America, prognosis, epidemiology, immunology, treatment

Latin America is a geographic region that shares cultural and genetic characteristics that may influence the susceptibility of its population to various diseases, including acute lymphoblastic leukemia (ALL), the most common form of childhood cancer, and acute promyelocytic leukemia (APL), which is also frequently observed among Hispanic populations (1). Notably, childhood ALL in Latino and Hispanic populations is associated with a lower frequency of gene rearrangements linked to favorable outcomes, alongside higher frequencies of gene patterns associated with poorer prognosis. Unfortunately, this corresponds with lower survival rates. It’s important to recognize that the exact causes of leukemia are not fully understood, and only a limited number of risk factors have been identified (2–4).

This Research Topic is devoted to current and in-progress scientific knowledge on epidemiological, biological and clinical aspects of acute leukemias in children and their control in the Latin American region. “Childhood Leukemias in Latin America: Epidemiology, Causality, Novel Predictive Profiles and Therapeutic Strategies” includes 17 Original Research articles, 2 Reviews, 1 Methods and 1 Perspective article, providing a comprehensive overview of the advancements in some of the malignant diseases that have been extremely heterogeneous and a cause of long-term diseases in Latin America.

In this Research Topic, expert authors explore the epidemiology, causation, prevention, pathobiology, diagnosis, novel predictive profiles, and therapeutic strategies related to this significant health priority in Latin America, given the rising incidence and mortality rates at very early stages of treatment.

## Genetic, epidemiological, and socio-environmental factors contributing to childhood leukemias in Latin America

Recent studies illuminate the genetic, environmental, and socioeconomic factors contributing to the high incidence rates of childhood leukemia in Latin America (5). This Research Topic includes a review of the role of genetic variation and Indigenous American ancestry in the etiology of childhood leukemias. It also discusses future research directions aimed at enhancing our understanding of the disparities in ALL incidence and outcomes among children of Latin American origin, insights that are crucial for informing future precision prevention efforts (de Smith et al.).

One notable study examined the interaction between specific genetic variants related to glucocorticoid secretion and birth characteristics in relation to ALL risk, building on previous hypotheses that endogenous cortisol may influence the elimination of pre-leukemic clones formed *in utero*. This study underscores the potential role of genes regulating the hypothalamic-pituitary-adrenal (HPA) axis in leukemia development, warranting further validation of these findings (de Carvalho et al.).

Additionally, a population-based study from Puebla, Tlaxcala, and Oaxaca reports incidence rates consistent with trends observed in other Latin American regions, identifying peaks for both lymphoblastic and myeloid leukemias. Variations among subregions may be linked to social and ethnic factors, highlighting the urgent need for targeted research into the socio-environmental determinants of leukemia (Flores-Lujano et al.).

A spatial analysis in Greater Mexico City further elucidates the potential environmental influences on leukemia incidence. Significant clustering of ALL cases is observed, with a relative risk indicating heightened incidence in identified clusters. Proximity to electrical installations and petrochemical facilities suggests that environmental factors contribute to leukemia risk, emphasizing the necessity for further investigation and preventive measures in high-risk areas (Duarte-Rodriguez et al.). Moreover, in another research ALL clusters potentially associated with carcinogenic sources, leading to higher risks of poor outcomes were identified (Calderon-Hernández et al.).

Finally, two large case-control studies examine the impact of maternal diet during pregnancy on the risk of childhood leukemias in infants (Mérida-Ortega et al.; Pérez-Saldívar et al.).

## Advancements in biomarker identification and risk stratification

A study featured in this Research Topic employs comprehensive profiling tools to identify unique biomarkers and microenvironment characteristics, advocating for early and personalized risk stratification. This approach has the potential to significantly enhance treatment strategies and improve patient outcomes. The focus on patient stratification and risk prediction has provided valuable insights, particularly regarding a subtype of ProB-ALL in Mexican children from vulnerable regions, which is linked to a higher risk of measurable residual disease (MRD) (Romo-Rodriguez et al.).

In another study, the authors characterize the systemic immunological profile of children undergoing treatment for B-ALL, evaluating various cell populations, chemokines, and cytokines as potential biomarkers for clinical follow-up. Given the challenges of monitoring immune cells and molecules, peripheral blood biomarkers offer a valuable alternative for tracking disease progression. As treatment advances, significant changes in the immunological profile are observed, highlighting a complex and dynamic immune response during induction therapy (Carvalho et al.).

Additionally, a systematic review underscores the role of mTOR activity as a predictive marker (Cuéllar-Mendoza et al.). Furthermore, the evaluation of genetic and clinical factors contributing to poor outcomes and hematological toxicity in pediatric patients with ALL from different regions of Latin America is presented (Escalante-Bautista et al.; Moreira et al.; Duffy et al.).

## Enhancing diagnosis and treatment for children with acute leukemias in Latin America: a multicenter perspective

Various multicenter collaboration strategies are outlined to enhance the diagnosis, treatment, and follow-up of children with acute leukemias (Friedrich et al.; López-Aguilar et al.). The National Project for Research and Incidence of Childhood Leukemias (PRONAII) in Oaxaca, Puebla, and Tlaxcala has made significant progress in improving diagnostic and follow-up capabilities in socioeconomically vulnerable regions of Mexico. This initiative fosters interdisciplinary collaboration and enhances the quality of care through advanced diagnostic techniques, including immunophenotyping and MRD studies. The success of PRONAII demonstrates the effectiveness of integrated strategies in reducing mortality and improving care in these regions (Núñez-Enriquez et al.).

An examination of long-term treatment experiences for children with APL at a tertiary hospital reveals the effectiveness of the IC-APL 2006 protocol over a 14-year period. Despite some therapy-related complications, this protocol has achieved high survival rates, reinforcing the notion that APL is curable with appropriate treatment. However, this success also highlights ongoing challenges related to resource availability in Latin America, underscoring the need for sustainable solutions to ensure consistent access to critical medications like arsenic trioxide (Murillo-Maldonado et al.). Additionally, the results of treatment in pediatric patients in resource-poor geospaces are reported (Gallardo-Pérez et al.).

A perspective article addresses the challenges of implementing immunotherapy in Mexico and other Latin American countries, particularly concerning CAR-T cell therapy, which shows promise for treating relapsed or refractory hematological malignancies (Bustamante-Ogando et al.).

## Integrating technology and non-pharmacological interventions

The investigation into non-pharmacological interventions revealed the transformative potential of Virtual Reality (VR) in enhancing the quality of life for children undergoing maintenance treatment for ALL. Through a crossover protocol involving 20 young patients, VR has proven effective in significantly reducing anxiety and modifying the perception of treatment duration. This promising approach highlights the value of integrating advanced technologies into pediatric care to alleviate the challenges faced by young patients during prolonged medical treatments (Velasco-Hidalgo et al.).

## Conclusion and proposal

Of note, this Research Topic highlights both the advancements and persistent challenges in pediatric leukemia care across Latin America. Significant progress has been made in symptom management, risk prediction, and treatment efficacy. However, disparities in resource access and socio-economic factors unfortunately remain. To further advance pediatric leukemia care in the region, we propose the following initiatives:

1. Expand technological integration: Increase the use of non-pharmacological tools, such as VR, to amplify patient care and accessibility.
2. Improve risk stratification: Develop region-specific predictive models according to genetic and environmental local identity factors, for the sake of precision treatment strategies.
3. Strengthen resource distribution: Create a regional network to improve the availability and equitable distribution of essential medications, promoting international cooperation to ensure a steady supply of critical treatments.
4. Promote preventive actions: Launch educational campaigns on maternal nutrition and collaborate with community organizations to implement primary and secondary preventive strategies against leukemia.
5. Foster regional and global collaboration: Build stronger partnerships between Latin American institutions and global research communities to facilitate knowledge exchange and resource sharing to fight childhood mortality.
6. Investigate environmental and socioeconomic disease determinants: Conduct further exhaustive research into socio-environmental factors inducing leukemia incidence and implement targeted interventions, accordingly.
7. Bring cutting-edge diagnostic and monitoring technology to vulnerable or remote regions to serve marginalized populations and enshrine the universal right to the benefits of inclusive and accessible science.

By implementing these strategies, we will contribute to narrowing the gap in disparities and improving the quality of unified care in Latin America, to ultimately improve the outcomes of all children suffering from leukemia. Regional commitment to innovation, population-oriented research, and harmonization of comprehensive and equitable management will be essential to ensure the best care and optimal support. This Research Topic illustrates the complementary fundamental, applied and clinical science being conducted by the local community.
